# Septic Shock Secondary to a Pyogenic Liver Abscess Following Complicated Appendicitis

**DOI:** 10.7759/cureus.18359

**Published:** 2021-09-28

**Authors:** Adio Kokayi

**Affiliations:** 1 Intensive Care Unit, University College Hospital, London, GBR

**Keywords:** abscess, pyogenic liver abscess, : liver abscess, appendicitis, sepsis, septic shock

## Abstract

Pyogenic liver abscesses (PLAs) are a rare condition in North America and Europe and, rarer still, the cause of septic shock. This case report will describe the rare occurrence of a PLA producing septic shock in a 36-year-old male residing in the United Kingdom following a case of complicated appendicitis.

The patient presented to the emergency department (ED) with a three-week history of intermittent loose stools, cramping abdominal pain, recurrent fevers, a heart rate of 111 beats per minute, a blood pressure of 94/58 mmHg, and a fever of 40.1 degrees Celsius. Despite prompt broad spectrum antibiotic administration and three liters of fluid resuscitation, the patient remained shocked which led to an ICU admission.

A CT scan prior to transfer found a 7 cm x 6 cm x 6 cm lesion representing a liver abscess (LA) as well as gross inflammatory change affecting the distal small bowel. The LA was managed through insertion of a percutaneous drain under ultrasound guidance performed by the interventional radiology team, as well as ongoing IV antibiotics. Following growth of the gut commensal *Streptococcus constellatus* from the abscess fluid culture, a colonoscopy was performed which found a severely distorted and inflamed terminal ileum with an impassable stricture, raising not only the suspicion of appendicitis but also Crohn’s disease.

Following the colonoscopy, after a total of 10 days admission, the patient was allowed to go home with a four-week course of oral co-amoxiclav. After discharge, the patient’s case was discussed in the gastroenterology inflammatory bowel disease (IBD) multi-disciplinary team meeting due to concerns raised about possible Crohn’s disease from the admission CT and following colonoscopy findings. Given the absence of relevant IBD symptoms, a reassuring outpatient MRI small bowel scan (found considerable resolution of the right iliac fossa inflammatory process) and a fecal calprotectin of 29 four months post discharge (normal=0-51 μg/g), it was concluded the terminal ileum changes were most likely accounted for by a complicated course of appendicitis. When reviewed in a telephone clinic 10 weeks post discharge, he was found to have no persistent gastrointestinal (GI) symptoms and was subsequently discharged.

This case highlights the importance of comprehensive imaging and colonoscopy in the work up of those patients with PLAs with no otherwise evident precipitating factor.

## Introduction

Pyogenic liver abscesses (PLAs) are a rare condition in North America and Europe [1-2]. Prior to early source control with antibiotic agents, historically these most frequently occurred secondary to diverticulitis, appendicitis, or other intra-abdominal infections [3]. They now most commonly occur secondarily to biliary tract diseases such as stones, strictures, or malignant obstruction which allow direct extension of infectious material into the liver due to blockage of biliary outflow [4].

This case report will describe the rare occurrence of a PLA producing septic shock in a 36-year-old male residing in the United Kingdom following a case of complicated appendicitis.

## Case presentation

The 36-year-old male presented to the emergency department (ED) of a London hospital with a three-week history of intermittent loose stools, cramping abdominal pain, and fevers. On the day of admission, the patient developed rigors and a headache with blurred vision which led him to attend hospital. Notably, he collapsed in the waiting room following which he was transferred to a resuscitation bay.

This young male who works in IT had no past medical history, took no regular medications, and had no relevant travel or family history. He was a non-smoker and reported no alcohol consumption in the month coming up to his admission. He also had no history of consumption of illicit drugs.

On assessment, he was noted to have a heart rate of 111 beats per minute (BPM), a blood pressure of 94/58 mmHg, and a fever of 40.1 degrees Celsius. His oxygen saturations were 100% on room air and his respiratory rate was 24 BPM.

On general examination the patient was noted to be pale. Respiratory examination found clear lung bases with good bilateral air entry. Examination of the cardiovascular system consisted of normal heart sounds, warm peripheries, and a capillary refill of less than two seconds. Right upper quadrant tenderness was noted during abdominal examination though his abdomen was soft with no signs of peritonism. No hepatomegaly was noted, bowel sounds were normal, and a digital rectal examination was unremarkable.

Investigations

A venous blood gas taken on admission to the ED revealed a lactate of 6.3 mmol/L, a base excess of -5.8 mmol/L, and a pH of 7.39 in keeping with the developing clinical picture of sepsis. The patient’s blood tests later revealed deranged liver function with raised inflammatory markers also. The tests found a C-reactive protein level (CRP) of 322 mg/L, white cell count of 5.01 x 109/L, neutrophils of 4.86 x 109/L, and eosinophils of 0.00 x 109/L. Liver function tests showed an alkaline phosphatase (ALP) of 193 IU/L, alanine transaminase (ALT) of 76 IU/L, bilirubin of 26 μmol/L, and albumin of 35 g/L. Renal bloods notably found a potassium of 2.9 mmol/L as well as an acute kidney injury (creatinine=149 mmol/L, urea=4.5 mmol/L, estimated glomerular filtration rate - eGFR=48 mL/min/1.73m², sodium=132 mmol/L). A rapid coronavirus polymerase chain reaction (PCR) test was also negative.

**Figure 1:**
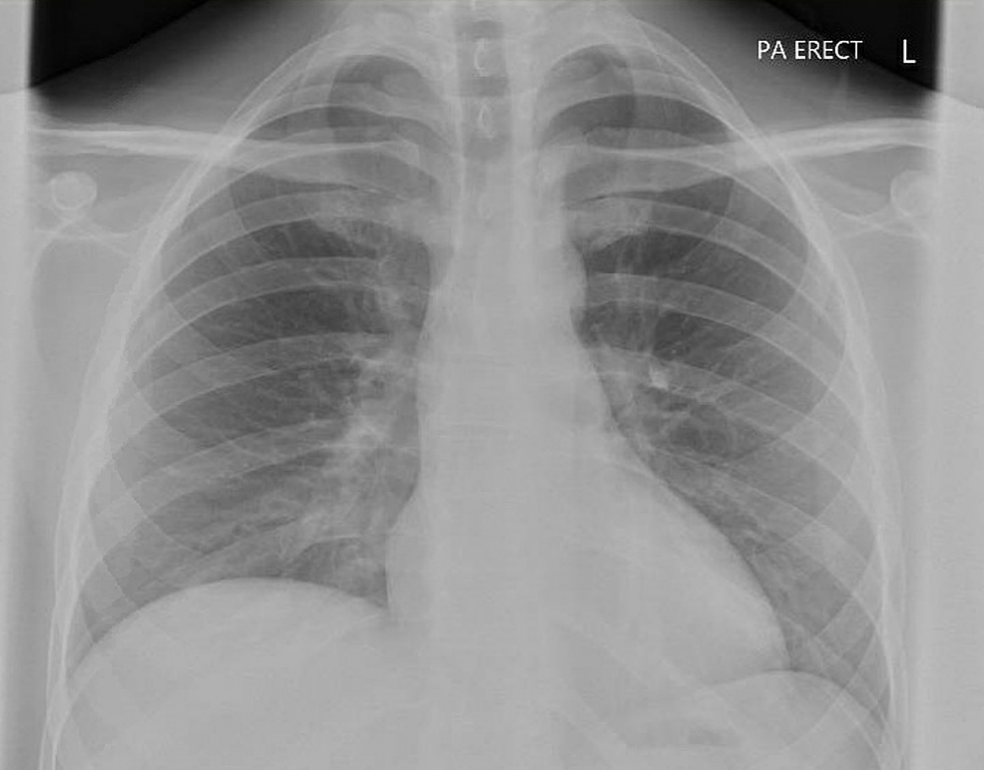
Admission chest X-ray.

**Figure 2:**
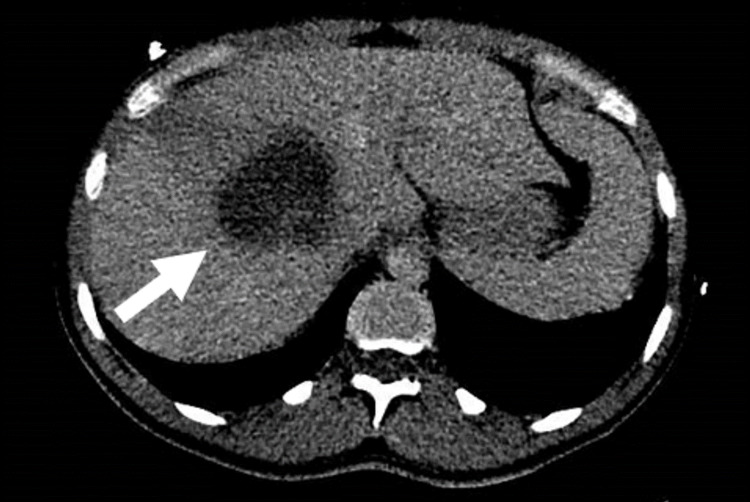
Liver abscess CT image.

In the ED the patient also underwent imaging with a plain chest radiograph and CT scanning (CT abdomen and pelvis with contrast, non-contrast CT Head, CT pulmonary angiogram). The patient’s chest X-ray (Figure 1) demonstrated clear lung fields with no effusion or pneumothorax, a normal mediastinum, and no cardiomegaly. CT scanning of the abdomen and pelvis found a central lesion of low attenuation measuring 7 cm x 6 cm x 6 cm which demonstrated no arterial enhancement and was reported as most likely representing a LA (Figure 2). In addition, gross inflammatory change affecting a cluster of the small bowel beneath the cecum was noted and was thought to represent appendicitis or terminal ileitis. Gall bladder swelling was noted, though there was no sign of cholecystitis. There were normal appearances of the pancreas, spleen, and kidneys and no abdominal or pelvic free fluid.

**Figure 3:**
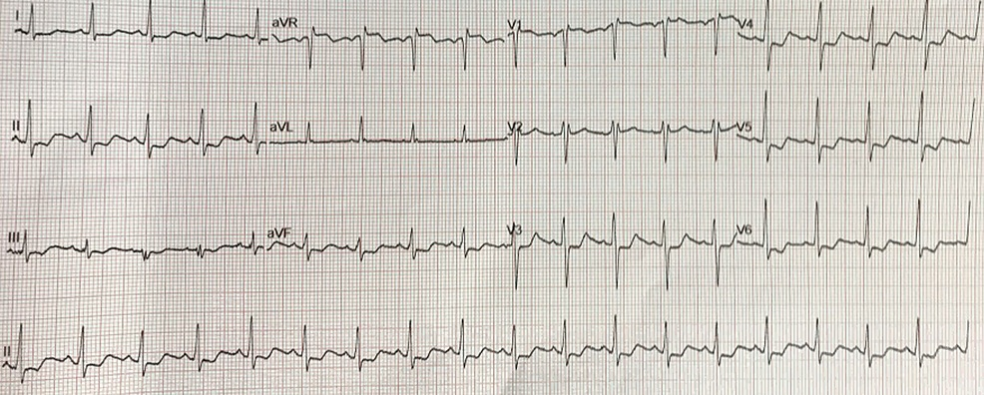
Admission ECG. ECG, electrocardiogram

**Figure 4:**
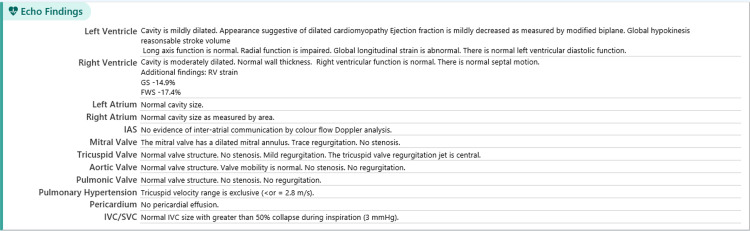
Echocardiogram report.

The patient was also reviewed by the hospital’s cardiology team during his admission. This specialist review was requested due to the finding of ST depression and sinus tachycardia on his admission electrocardiograms (ECGs) (Figure 3). Admission blood tests also revealed an elevated troponin T level of 40 ng/L which had risen to 59 13 hours later. This was followed by a transthoracic echocardiogram (TTE) which found a mildly dilated, globally hypokinetic left ventricle, mild left ventricular impairment and moderately dilated right ventricle but good longitudinal and radial function and trace mitral and tricuspid regurgitation (Figure 4). 

The evening following the patient’s admission he underwent ultrasound-guided percutaneous drainage of the abscess under the interventional radiology team. Some 100 mL of frank pus was aspirated which was then sent for microbiological assessment with a 12 Fr pigtail drain left in situ. A repeat ultrasound two days later revealed resolution of the collection, following which the drain was removed. Three days following sampling, the abscess fluid culture found growth of Streptococcus constellatus. Otherwise stool bacterial PCR, *Clostridium difficile* testing, ova/cysts/parasite microscopy, amoebic serology, and modified Ziehl-Nielsen testing for Cryptopsoridium were negative.

**Figure 5:**
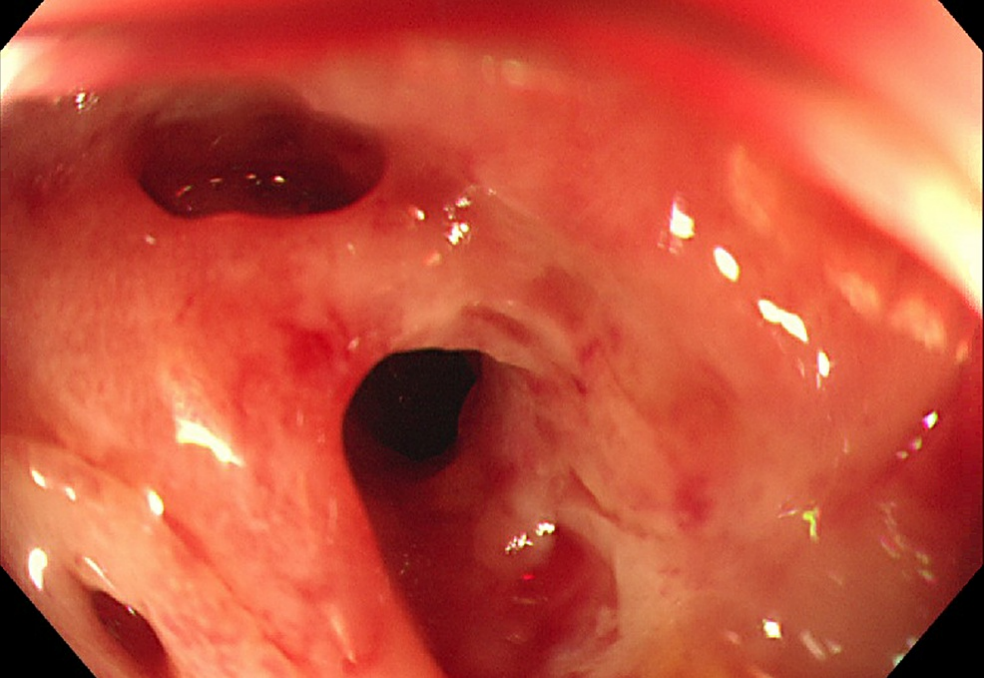
Colonoscopy image.

Given its role as a part of normal gut flora and suspected translocation from the bowel leading to the LA, a colonoscopy was arranged under the gastroenterology team for visual evaluation of the gastrointestinal (GI) tract. This found a severely distorted and inflamed terminal ileum with an impassable stricture (Figure 5). Terminal ileum biopsies taken at the time found fragments of ulcerated mucosa and superficial submucosa which showed granulation tissue with a heavy lymphoplasmacytic inflammatory infiltrate. The infiltrate displayed no atypical morphological features and no granulomas or microorganisms were seen.

Treatment

Despite fluid resuscitation with three liters of crystalloid fluid and IV metronidazole, the patient was persistently shocked with a blood pressure of 89/40 mmHg and a urine output of approximately 40 mL/h (weight=83 kg). This led to escalation of his care to the intensive care team and subsequent vasopressor support with noradrenaline. At this time, advice was sought from the microbiology team who advised adding IV cefuroxime to his regular regime alongside metronidazole, as well as a single dose of IV gentamicin.

By day three of the patient’s admission he had no further requirement for vasopressor support, his acute kidney injury had resolved (urea=4.5 mmol/L, creatinine=81 mmol/L, eGFR=>90 mL/min/1.73m²) and his vital signs had normalized (afebrile, heart rate=89 BPM, blood pressure=108/53 mmHg). Given the patient’s stable condition, he was stepped down from the ICU to a normal ward environment under the care of the infectious diseases team.

Outcome and follow-up

Following the colonoscopy, after a total of 10 days admission, the patient was allowed to go home with a four-week course of oral co-amoxiclav. On outpatient clinic review three weeks later by the infectious diseases team, the patient reported no persisting abdominal symptoms. Examination was also normal. Bloods taken on the day also demonstrated resolution of his raised inflammatory markers with good kidney function (white cell count=6.00 x 109/L, CRP=17.0 mg/L, creatinine=84 mmol/L). Deranged liver function persisted to some extent but had dramatically improved when compared to tests taken at discharge.

Two months after discharge, the patient’s case was discussed in the gastroenterology inflammatory bowel disease (IBD) multi-disciplinary team meeting due to concerns raised about possible Crohn’s disease from the admission CT and following colonoscopy findings. Given the absence of relevant IBD symptoms, a reassuring outpatient MRI small bowel (found considerable resolution of the right iliac fossa inflammatory process) and a fecal calprotectin of 29 four months post discharge (normal=0-51 μg/g), it was concluded the terminal ileum changes were most likely accounted for by a complicated course of appendicitis. When reviewed in a telephone clinic 10 weeks post discharge he was found to have no persistent GI symptoms and was subsequently discharged.

Given the patient’s abnormal admission ECG and TTE findings, outpatient follow-up with the cardiology department was also arranged. While the inpatient TTE found a mildly impaired ejection fraction of 47%, a cardiac MRI (adenosine stress protocol) carried out 10 weeks after discharge found normal pump function. However, there was persistent mild left ventricular dilatation (end diastolic volume=202 mL; normal range=99-199 mL). A telephone clinic consultation at four months following discharge revealed the patient had no cardiac symptoms and was undertaking four exercise sessions weekly (including two 10 km runs). He was discharged from regular clinic review with a follow-up echocardiogram scheduled for three years’ time.

## Discussion

Defined as a microbial contamination of liver parenchyma leading to a pus filled hepatic collection, a LA is typically pyogenic or amoebic, though rarely can develop due to fungal infections [5]. Whereas PLAs make up the majority of cases in western nations, in South East Asia and Africa amoebic abscesses are most common [6]. While higher rates of infection occur in some regions of the world, in Europe and North America PLAs are uncommon [1-2, 7]. In a population based study of the United States, Meddings et al. found a PLA incidence of 3.6 per 100,000 cases [8]. This retrospective study of almost 18,000 cases between 1994 and 2005 also found that males were most often affected (59.9%) while the age group 65-85 had the highest rate of PLA infection.

The PLAs develop through three mechanisms. One such is spread of bacteria from an infection or collection from a source drained by the portal venous system (intra-abdominal), leading to its transfer to the liver. Furthermore, biliary tract diseases (e.g., strictures, malignancy, gallstones) which cause biliary obstruction allow direct extension of infectious material to the liver and thus result in abscess formation. Finally, direct hematogenous spread of systemic bacteria, caused by bacterial endocarditis or periodontal infections for example, are recognized causes of PLAs [4]. Significant risk factors that promote the occurrence of these processes are immunosuppression, liver transplants, diabetes mellitus, biliary procedures, and intra-abdominal cancers [9].

Both Gram-negative and Gram-positive organisms are involved in PLAs. Gram-negative infections are commonly caused by *Klebsiella pneumonia*, *Escherichia coli*, and *Pseudomonas aeruginosa*, while Gram-positive organisms most often include *Streptococcus* and *Staphylococcus* [9]. The microbiological profile of PLAs demonstrates a well-documented geographical variation: in South-East Asian countries *Klebsiella* is the organism most frequently implicated in PLAs, whereas in Western countries, *Streptococcus* is most often noted [10].

The LAs typically present with abdominal pain and fever, with cough (due to pleural effusion and collapse of underlying lung parenchyma) and loose stools occurring less frequently [10]. As is the case for evaluation of most hepatic pathologies, ultrasound imaging is the modality of choice for the LA evaluation. Further imaging, such as CT or MRI, may be useful in diagnosing the abscess source, however [11].

In the treatment of PLAs, antibiotic therapy and abscess drainage is essential. Antibiotic therapy must be broad spectrum before sample cultures allow them to be rationalized, with regimes often including an aminoglycoside (such as gentamicin) and metronidazole. In cases where a *Staphylococcus* or *Streptococcus* is grown on a culture, a penicillinase-resistant penicillin or cephalosporin can be used. With regard to anti-amoebic therapy, treatment with metronidazole as a single agent is usually sufficient [11]. Drainage is most frequently achieved through percutaneous access (ultrasound or CT guidance), though laparoscopic and open procedures may be undertaken [12]. 

## Conclusions

This case demonstrates that in the assessment of a patient with a LA, endoscopic evaluation of the GI tract may be a key investigation in identifying and evaluating the precipitating cause. Furthermore, it has been clearly demonstrated that the management of a LA requires a multidisciplinary approach, with this patient’s case requiring expertise from gastroenterology, infectious diseases, intensive care, cardiology, and interventional radiology among others.

Finally, a consensus on the patient’s diagnosis (complicated appendicitis) could only be reached several months after his discharge. This highlights how significant diagnostic uncertainty is sometimes unavoidable. On these occasions, empirical treatment is the only option until this can be resolved.
